# Autosomal and mtDNA Markers Affirm the Distinctiveness of Lions in West and Central Africa

**DOI:** 10.1371/journal.pone.0137975

**Published:** 2015-10-14

**Authors:** Laura D. Bertola, Laura Tensen, Pim van Hooft, Paula A. White, Carlos A. Driscoll, Philipp Henschel, Anthony Caragiulo, Isabela Dias-Freedman, Etotépé A. Sogbohossou, Pricelia N. Tumenta, Tuqa H. Jirmo, Geert R. de Snoo, Hans H. de Iongh, Klaas Vrieling

**Affiliations:** 1 Leiden University, Institute of Environmental Sciences (CML), PO Box 9518, 2300, RA Leiden, The Netherlands; 2 Leiden University, Institute of Biology Leiden (IBL), PO Box 9505, 2300, RA Leiden, The Netherlands; 3 University of Johannesburg, Department of Zoology, PO Box 524, Johannesburg, Johannesburg, Republic of South Africa; 4 Wageningen University, Resource Ecology Group, Droevendaalsesteeg 3a, 6708, PB Wageningen, The Netherlands; 5 Center for Tropical Research, Institute of the Environment and Sustainability, La Kretz Hall Suite 300, 619 Charles E. Young Dr. East, University of California Los Angeles, Los Angeles, CA, 90095–1496, United States of America; 6 Wildlife Institute of India, Dehradun, 248 001, Uttarakhand, India; 7 Panthera, 8 West 40th Street, 18th Floor, New York, NY, 10018, United States of America; 8 Sackler Institute for Comparative Genomics, American Museum of Natural History, 79th Street at Central Park West, New York, NY, 10024, United States of America; 9 Laboratoire d’Ecologie Appliquée, Université d’Abomey-Calavi, Champ de Foire 03 BP 1974, Cotonou, Benin; 10 Centre for Environment and Development Studies in Cameroon, University of Dschang, BP 410, Maroua, Cameroon; 11 University of Antwerp, Department Biology, Evolutionary Ecology Group, Groenenborgerlaan 171, 2020, Antwerpen, Belgium; University of York, UNITED KINGDOM

## Abstract

The evolutionary history of a species is key for understanding the taxonomy and for the design of effective management strategies for species conservation. The knowledge about the phylogenetic position of the lion (*Panthera leo*) in West/Central Africa is largely based on mitochondrial markers. Previous studies using mtDNA only have shown this region to hold a distinct evolutionary lineage. In addition, anthropogenic factors have led to a strong decline in West/Central African lion numbers, thus, the conservation value of these populations is particularly high. Here, we investigate whether autosomal markers are concordant with previously described phylogeographic patterns, and confirm the unique position of the West/Central African lion. Analysis of 20 microsatellites and 1,454 bp of the mitochondrial DNA in 16 lion populations representing the entire geographic range of the species found congruence in both types of markers, identifying four clusters: 1) West/Central Africa, 2) East Africa, 3) Southern Africa and 4) India. This is not in line with the current taxonomy, as defined by the IUCN, which only recognizes an African and an Asiatic subspecies. There are no indications that genetic diversity in West/Central Africa lions is lower than in either East or Southern Africa, however, given this genetic distinction and the recent declines of lion numbers in this region, we strongly recommend prioritization of conservation projects in West/Central Africa. As the current taxonomic nomenclature does not reflect the evolutionary history of the lion, we suggest that a taxonomic revision of the lion is warranted.

## Introduction

Identifying and describing patterns of mitochondrial (mtDNA) and nuclear genetic variation is a crucial component to fully understanding the evolutionary history of a species. High quality phylogeographic data that represent the underlying genetic complexity are important for taxonomy and contribute to designing effective conservation strategies. This is of particular importance for species such as the lion (*Panthera leo*) that occupy large geographic ranges within which disjunct populations may not allow for natural dispersal and gene flow. Increasing habitat fragmentation and variable anthropogenic factors have created a growing need to manage lions at the population level [1]. In addition, several recent publications have sparked the discussion whether the current taxonomic nomenclature for the lion is justified [2–4].

**Fig 1:**
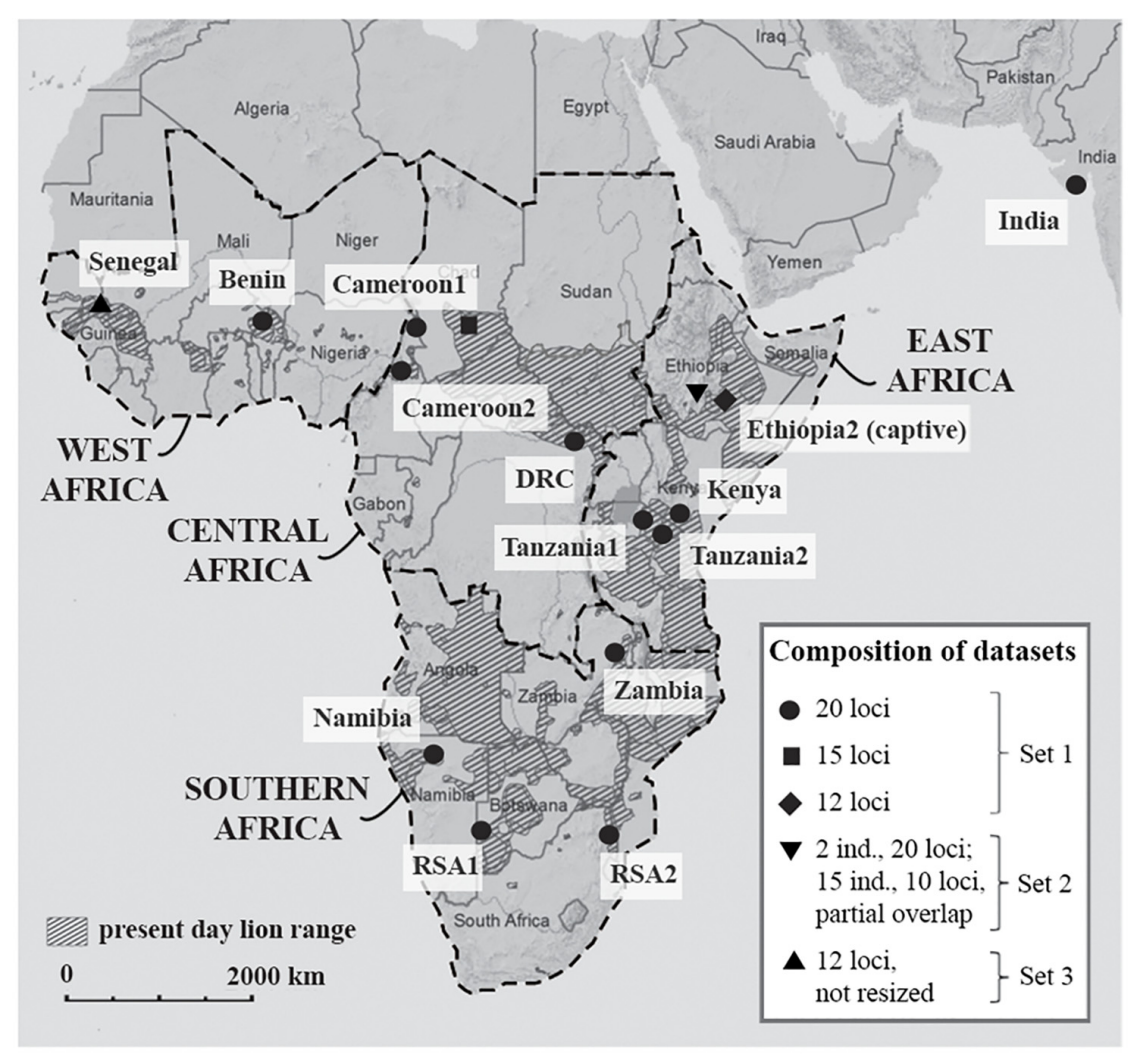
Map showing the location of the 16 lion populations included in the analysis. In the legend, the composition of the datasets and the number of included microsatellite loci is indicated. Lion range data from IUCN (2014). Region definitions from IUCN SSC Cat Specialist Group (2006a; b).

Two subspecies of lion are officially recognized by the IUCN, based on genetic data [5,6]: the African lion (*Panthera leo leo*), ranging throughout sub-Saharan Africa with the exception of dense rain forest, and the Asiatic lion (*Panthera leo persica*), which exists as a single population in the Gir forest, India. Although all African lion populations are considered as belonging to the African subspecies (*P*. *l*. *leo*), distinct subgroups have been recognized based on morphology [7,8] and genetics [2–5,9–12]. Analyses of morphometric data has led to the distinction of at least three extant clades (“subspecies”) on the African continent [7]. Lions from the northern part of their range further showed a relatively close relationship to the Asiatic subspecies [7,8]. This pattern was confirmed by phylogenetic analysis of mitochondrial haplotypes only, based on which lions in West/Central Africa were described as a genetically distinct group with a relatively close genetic relationship to the Asiatic subspecies [2–4] (region definitions from [13,14], see Fig 1). The genetic dichotomy that separates the West/Central African lion populations from East and Southern African populations has also been found in other large mammal species and is often reflected in their taxonomy including African buffalo (*Syncerus caffer*) [15,16], roan antelope (*Hippotragus equinus*) [17], hartebeest (*Alcelaphus buselaphus*) [18,19], giraffe (*Giraffa camelopardalis*) [20,21] and cheetah (*Acinonyx jubatus*) [22,23].

Due to the genetic differentiation within the African lion and the nested position of the Asiatic lion subspecies within the West/Central Africa clade based on mtDNA, the current taxonomic division is challenged [2–4]. However, mtDNA is a single, non recombining locus in the maternal lineage and does not permit the detection of admixture events and sorting at multiple loci as may occur in autosomal markers. Therefore, the observed pattern in mtDNA data may not adequately depict the underlying genetic complexity. Autosomal data are needed to corroborate the topology based on mtDNA, since conflicting patterns between phylogenies based on mtDNA and phylogenies based on autosomal markers have been described in several other species [24–29]. Most commonly a monophyletic pattern is detected in the mtDNA, but is not supported, or is contradicted, by phylogenies based on autosomal loci. This is often explained by incomplete lineage sorting, as coalescence time in mtDNA is four times shorter than in autosomal markers. Since lineage sorting during the process of coalescence has a random nature, this could also lead to an ‘incorrect’ gene tree by mtDNA markers if populations’ divergences were closely spaced in time. Female philopatry is another strong contributing factor in mtDNA trees. As gene flow in lions is biased towards the male sex [30,31], gene trees based on autosomal markers may show less discrete groups. This argument has been used by Antunes *et al*. (2008) to explain incongruent patterns in their lion data based on mtDNA and autosomal markers. Taxonomic revisions have potentially far-reaching ramifications with regard to management (e.g., CITES, USFWS, IUCN) and, therefore, should be approached cautiously. Ideally, proposed revisions should be supported by a combination of biogeographic, mtDNA and autosomal DNA, and morphological data.

In this study, we analyzed 20 microsatellite loci for lions from thirteen wild populations, one of which is located in West Africa (Benin) and four in Central Africa (Chad, DRC and two from Cameroon). Furthermore, we included microsatellite data from another West African population in Senegal and from two distinct zoo populations of Ethiopian lions representing the region where the two major genetic lineages (i.e., West/Central Africa and East/Southern Africa) may connect. To compare the phylogenetic clusters derived from the microsatellite data and to check for congruence with previously published patterns, we included data from 1,454 base pairs (bp) of the mitochondrial DNA for each sampling location. Using this approach, we are aiming to contribute to the ongoing discussion about lion taxonomy by answering four questions: 1) Do autosomal data support previously described phylogenetic groupings in the lion in general and the distinct position of the West/Central African lion in particular?, 2) Can an effect of sex-biased gene flow be detected?, 3) How genetically distinct are the sampled populations, at both the continental and regional scales, and do levels of genetic diversity vary amongst regional subdivisions, with a special focus on West/Central Africa? and 4) Are there signs for reduced genetic diversity in particular lion populations with an emphasis on West/Central Africa? Our study is the first to include multiple lion populations from West/Central Africa, using both autosomal and mtDNA markers in a phylogenetic context covering the entire current geographic range of the lion.

## Material and Methods

We processed a total of 48 samples from eight populations, including one population from West Africa (Benin), four populations from Central Africa (two from Cameroon, one from Chad and one from DRC), two populations from East Africa (Ethiopia2 (captive) and Kenya) and one population from Southern Africa (Zambia). Except for Ethiopia2, all samples originated from free-ranging lions, with no known history of anthropogenic introductions of lions from other populations. Samples were collected in full compliance with specific permits (CITES and permits related to national legislation in the countries of origin). Details on permits, sample storage, DNA extraction, polymerase chain reaction (PCR) amplification, fragment analysis and quality control are given in S1 File. See S1 Table and S2 Table for used loci and primer information. All microsatellite allele length data are given in S2 File.

Generated microsatellite data were supplemented by published data for the same 20 loci from another six populations [32], together summarized as Dataset 1. Dataset 2 [12] consists of all 15 samples from Ethiopia1 (captive) with ten analyzed loci, of which six are overlapping with our dataset. For two samples from Ethiopia1, all 20 microsatellites were analyzed and added to Dataset 1. Dataset 3 (Panthera/AMNH) contains microsatellite data from 12 loci for seven lions from Senegal, which could not be resized to Dataset 1 and were therefore only included for calculation of diversity indices and bottleneck statistics (for details on permits and the processing of Senegal samples, see S3 File). An overview of datasets used in each analysis is provided in Fig 1 and Table 1.

**Table 1:** Overview of lion populations included in this study.

Set	Population	Area	Geographic Region	PopSize	N msat	N mtDNA	Source msat data
	**Benin**	Pendjari NP	West Africa	100	5	5	this dataset
	**Cameroon1**	Waza NP	Central Africa	20	9	9	this dataset
	**Cameroon2**	Bénoué Ecosystem	Central Africa	200	3	3	this dataset
	**Chad**	Zakouma NP	Central Africa	140	4	4	this dataset
	**DRC**	Garamba NP	Central Africa	175	7	6	this dataset
	**Ethiopia2**	Yemen Zoo	East Africa	(captive)	4	4	this dataset
**1**	**Kenya**	Amboseli NP	East Africa	60	7	7	this dataset
	**Tanzania1**	Serengeti NP	East Africa	3465	10	3	Driscoll et al., 2002
	**Tanzania2**	Ngorongoro CA	East Africa	53	10	1	Driscoll et al., 2002
	**Zambia**	Luangwa Valley	Southern Africa	750	9	9	Driscoll et al., 2002
	**Namibia**	Etosha NP	Southern Africa	455	10	2	Driscoll et al., 2002
	**RSA1**	Kalahari-Gemsbok NP	Southern Africa	350	10	2	Driscoll et al., 2002
	**RSA2**	Kruger NP	Southern Africa	1684	10	10*	Driscoll et al., 2002
	**India**	Gir forest NP	India	411	10	6	Driscoll et al., 2002
**2**	**Ethiopia1**	Addis Ababa Zoo	East Africa	(captive)	15	5	Bruche et al., 2012
**3**	**Senegal**	Niokolo Koba NP	West Africa	15	7	7	Panthera/AMNH

PopSize: population size according to the most recent estimate in Riggio et al. (2012) for the African populations, except for Zambia: Paula White (personal communication); estimate for the Indian population from [56]

N msat: number of sampled individuals for microsatellite analysis

N mtDNA: number of sampled individuals for mtDNA analysis.

* mtDNA and microsatellite data are not from the same samples.

STRUCTURE 2.3.3 [33] was used for assessing population structure in Dataset 1 with unknown loci scored as missing data. Simulations were run assuming the admixture model with correlated allele frequencies. Ten runs were performed for K = 1 to K = 8, using 10,000,000 permutations and a burn-in period of 1,000,000. To check the assignment of Ethiopia1 to any of the clusters identified by STRUCTURE, we included the two Ethiopian samples for all 20 microsatellites. Structure Harvester [34] was used to determine the most likely number of clusters, following the ΔK method [35]. CLUMPP was used to combine replicate runs and avoid label switching [36]. Clustering of individuals was further assessed by performing Principal Component Analysis (PCA) in GenAlEx 6.501 [37]. A neighbour-joining tree was created based on D_A_ distance in POPTREE2 using 1,000 bootstraps [38].

For each sampling location, a mitochondrial region of 1,454 bp that encompassed cytochrome B (cytB), tRNAThr, tRNAPro and part of the control region was included for a number of individuals (Table 1). Details on polymerase chain reaction (PCR) amplification and sequencing are given in S1 File. Sequences were deposited in GenBank and supplemented by sequences previously published by Bertola *et al*. (2011) (see S4 File for sequence data and S3 Table for accession numbers). Variable sites and nucleotide diversity were calculated using ARLEQUIN 3.5 [39]. For phylogenetic analysis, a haplotype network was created using the median-joining algorithm in Network 4.6.1.1 (www.fluxus-engineering.com). A repeat region of cytosines of variable length was excluded due to unknown homology (positions 1382–1393) and all remaining characters were included with equal weighting.

For AMOVA of Dataset 1, individuals for which all 20 loci were analyzed were included as either 1) without an indicated substructure (as all 1 group), 2) following IUCN classification (Africa; Asia), 3) following a North/South division as was indicated from the haplotype network, or 4) using the four groups identified by STRUCTURE (West/Central Africa; East Africa; Southern Africa; India). Isolation By Distance (IBD) was assessed by correlating geographic to genetic distances and using a Mantel’s permutation test with 999 permutations, as implemented in GenAlEx 6.501 [37]. In addition, AMOVA and IBD analysis were performed on a regional level, using the regions as indicated above (Africa; North; South; West/Central Africa; East Africa; Southern Africa). Pairwise F_ST_ and Nei’s genetic distances were computed with GenAlEx 6.501 [37] for microsatellite data and with ARLEEQUIN 3.5 for mtDNA data [39].

The average number of alleles per locus (Na) was calculated using ARLEQUIN 3.5 [39]. Private allelic richness (A_p_) was calculated with HP-Rare 1.1 [40] including statistical rarefaction to compensate for different sample sizes. GenAlEx 6.501 [37] was used to calculate observed (Ho) and unbiased expected heterozygosity (uHe) [41]. To obtain insights into the risk of emergent inbreeding, F_IS_ per population was calculated in FSTAT [42] and the occurrence of recent bottlenecks was evaluated by using the program Bottleneck [43,44]. The Bottleneck test is based on the theory that during a bottleneck the allele numbers are reduced faster than the heterozygosity, leading to an excess of heterozygosity compared to the expected heterozygosity under the mutation-drift equilibrium. The program was run for 10,000 iterations, using the stepwise mutation model (SMM). Significant (P<0.05) results from the Wilcoxon signed-rank test were scored, as this test proved to be the most powerful and robust when used with few (<20) polymorphic loci [44].

## Results

**Fig 2:**
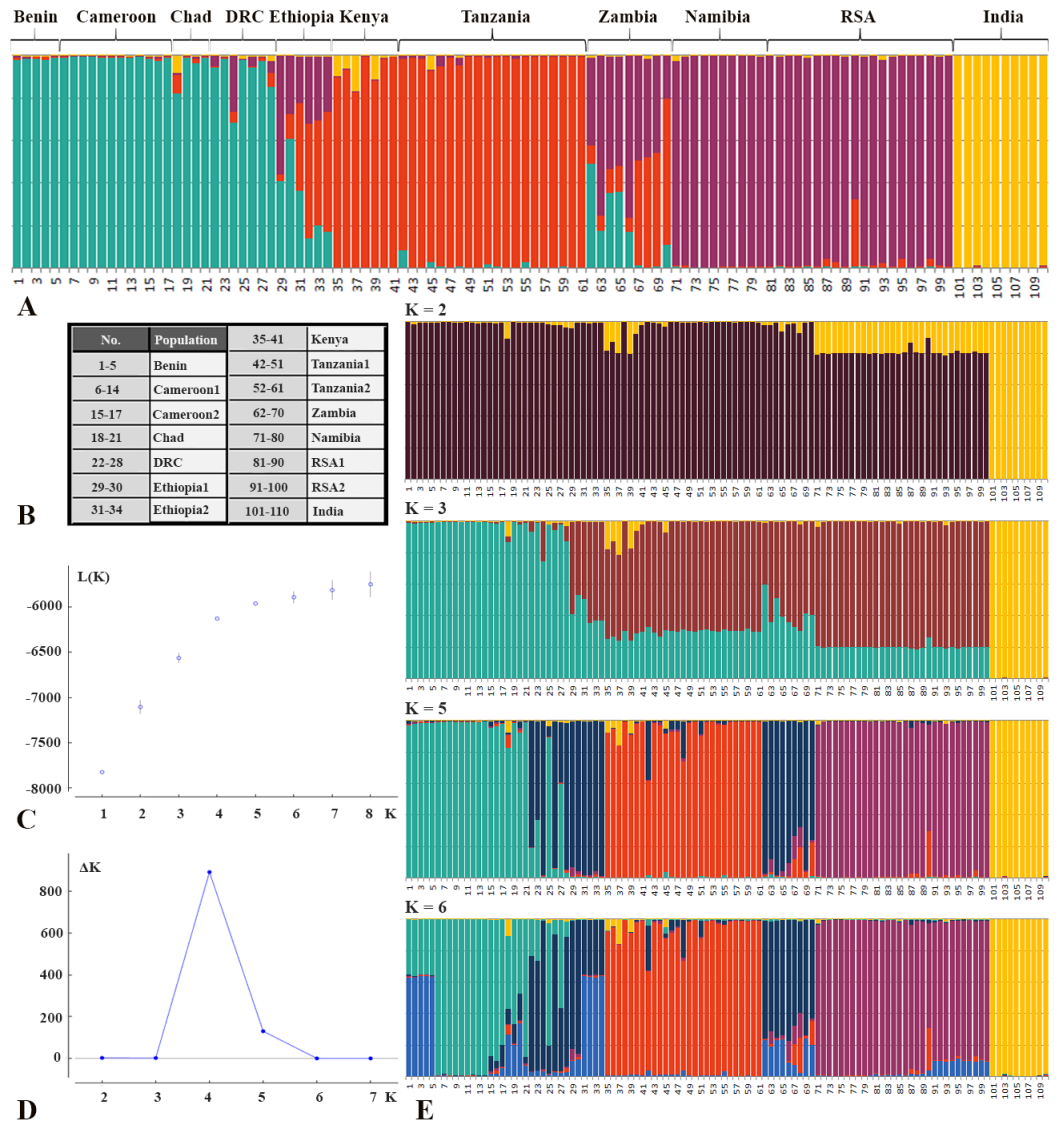
Results of STRUCTURE analysis based on 20 microsatellite loci of 15 lion populations (Dataset 1 + 2 individuals from Ethiopia1). A: representation of assignment values found by STRUCTURE, using K = 4; B: Overview of included populations; C: Plot indicating mean log likelihood Ln (P(X|K); D: plot indicating ΔK values as a function of the number of genetic clusters (K), in which ΔK = mean(|L”(K)|)/sd(L(K)); E: Representation of assignment values found by STRUCTURE, using K = 2. K = 3, K = 5 and K = 6.

**Fig 3:**
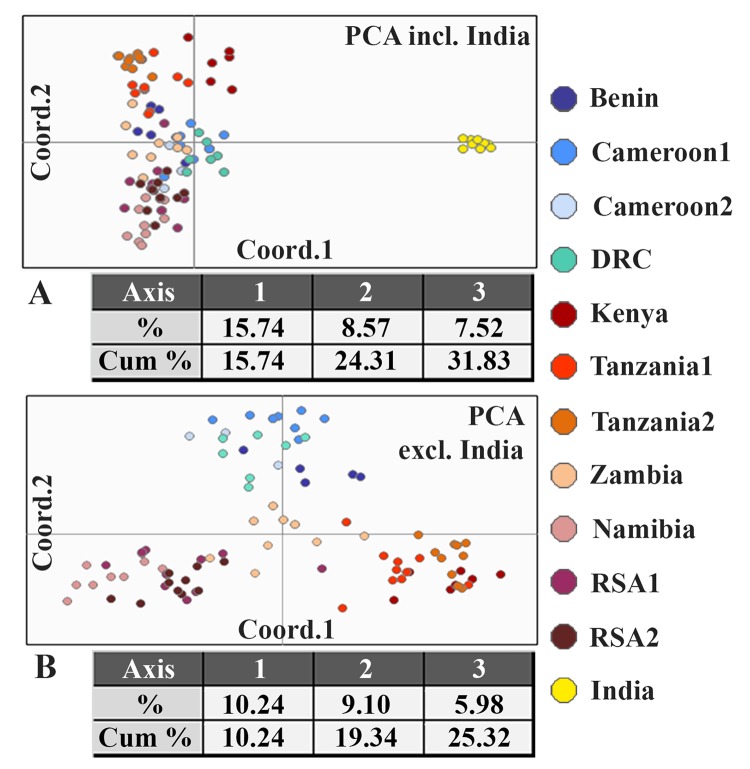
Results of PCA based on 20 microsatellite loci of lion populations. A: results of PCA of 12 populations (Dataset 1, excluding Chad and Ethiopia2), shown in a two-dimensional plot and a table indicating the percentage and the cumulative percentage explained by the first three axes; B: Results of PCA of 11 populations, excluding India.

Based on the STRUCTURE results of Dataset 1, Structure Harvester identified that the observed genetic structure is best described by four clusters representing the following geographic areas: 1) West/Central Africa, 2) East Africa, 3) Southern Africa and 4) India (Fig 2). There is no indication for a hierarchical structure, and forcing the program to identify a different number of clusters leads to an artificial clustering characterized by heavy admixture and results not in line with suggested evolutionary history derived from other data. Individuals from Chad are part of the West/Central Africa cluster. The Ethiopian lions show affiliation either to West/Central Africa, admixed with Southern Africa (Ethiopia1) or to East Africa, admixed with Southern Africa (Ethiopia2). The Zambia population shows a substructure as a result of admixture. All Zambian individuals are partially assigned to the Southern Africa cluster, and depending on the individual, either to West/Central Africa, or to East Africa. The admixed signal of the Zambia population is also visible by the central position in the plot of the first two axes of the PCA when India is excluded (Fig 3B). STRUCTURE runs were repeated excluding Indian genotypes, since PCA illustrated the effect of India (Fig 3A) and it is known that STRUCTURE has the tendency to force clustering in inappropriately small number of clusters under certain circumstances [45]. This may be the case if a single population contains markedly less genetic diversity which drives the program to place all remaining populations into a single cluster thereby providing a result which does not properly reflect the evolutionary history [45]. These analyses did not lead to a difference in clustering of the remaining individuals and the same three groups were identified within Africa (data not shown).

**Fig 4:**
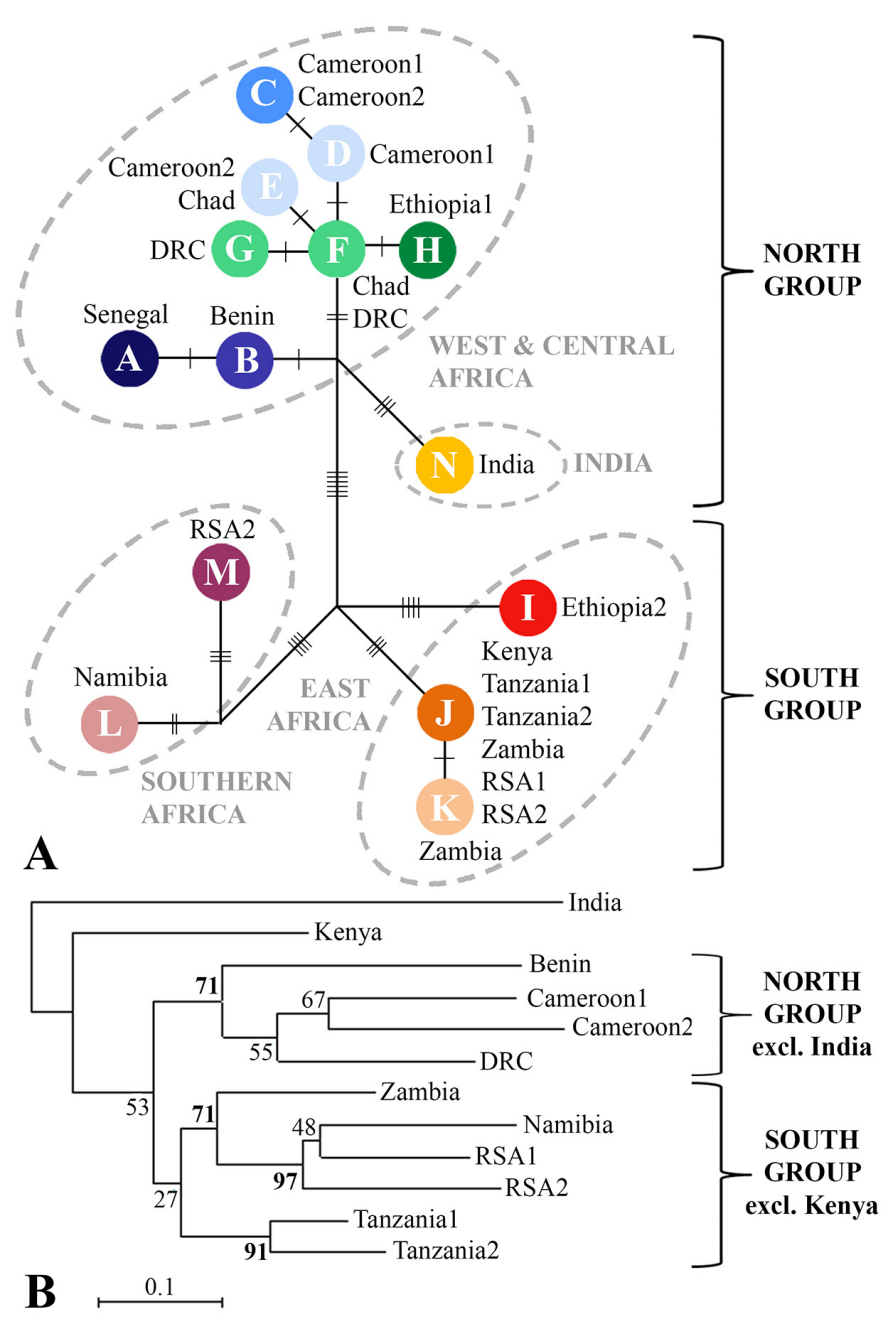
Relationship between populations of lions based on mtDNA data and on 20 microsatellite loci. A: Haplotype network based on median-joining algorithm in Network; B: Phenetic tree based on D_A_ genetic distance of microsatellite data of 12 lion populations.

A total of 87 sequences of 1,454 bp were analyzed. Nucleotide diversity (π) was 0.102. Based on 43 polymorphic sites, 15 different haplotypes were distinguished. The haplotype network (Fig 4A) and the neighbour-joining tree (Fig 4B) based on the microsatellite data show a similar topology in which West/Central African lions are grouped together on a supported branch (bootstrap value >70) and East and Southern African lions are represented on two different supported branches (Fig 4B). A basal split into a North group (West/Central Africa and India) and a South group (East Africa and Southern Africa) is most clearly visible in the haplotype network, as the clustering of East Africa and Southern Africa on a South branch in the phenetic tree has only limited support. Furthermore, Kenya and India both have a basal and unresolved position in the tree.

Results from AMOVA of the microsatellite data show that using the clusters identified by STRUCTURE to assign populations to four groups resulted in a relatively high percentage of the molecular variance being attributed to among-groups for microsatellite data (17.4%) and mtDNA data (52.3%) (S4 Table). While in the microsatellite data the highest percentage (29.6%) of molecular variance in among-groups variance is attributed to the split between Africa and Asia, i.e. between the two subspecies, no molecular variance among-groups for the Africa/Asia division is found in the mtDNA data. In addition, following the basal split in a North group and a South group, AMOVA attributes 54.6% of molecular variance to among-groups variance for mtDNA data, but only finds 7.5% in among-groups variance when using microsatellite data. However, absolute percentages may be misleading, as within-population variance is very different amongst the used markers.

Mantel tests showed that the effect of isolation by distance is evident, both on the continental and the regional scale (summary and graphs in S5 Table). In regional analyses, the highest values for among-groups variance according to AMOVA and the highest numbers for the slope of the trend line in IBD are found in West/Central Africa (compared to the South group, East Africa or Southern Africa) suggesting strong isolation between these populations. Pairwise F_ST_ values ranged from 0.064 to 0.736 and were significant for all pairwise comparisons (50,000 permutations, P<0.05) (S6 Table). Within Africa, pairwise F_ST_ values ranged from 0.064 to 0.396. Nei’s genetic distance ranged from 0.196 to 2.193 for all lion populations and within Africa it ranged from 0.196 to 2.018 (S6 Table).

Diversity indices (S7 Table) show that the Indian population comprises the lowest number of microsatellite alleles per locus, smallest allelic range and the highest number of fixed alleles. In the Indian population, 75% of the loci are fixed while in all other populations at maximum 17% of the loci are fixed. Diversity indices were found to be relatively constant across the African populations; surprisingly West/Central Africa showed no clear signs of loss of genetic diversity. Four out of seven populations in West/Central Africa contained more than one haplotype (Cameroon1, Cameroon2, Chad, DRC), whereas this was only observed for two out of eight populations in East and Southern Africa (Zambia and RSA2). Observed and expected heterozygosity values further confirmed the low genetic diversity of the Indian population. F_IS_ values illustrated a significant heterozygosity excess in Benin (P<0.01) and Cameroon1 (P<0.01) and a significant heterozygosity deficiency in Zambia (P<0.01), RSA1 (P<0.05) and Ethiopia1 (P<0.05). Results of the bottleneck analysis showed that there was a significant excess of heterozygotes found in Cameroon1 (P<0.01), Kenya (P<0.05) and Ethiopia1 (P<0.05), possibly indicating a recent reduction in population size.

## Discussion

Here we describe the distinct position of lions in West/Central Africa, compared to other populations across the lion’s current geographic range, based on phylogenetic analyses of microsatellite and mtDNA datasets. Moreover, we assessed levels of genetic diversity across different geographic scales to detect signs of low genetic diversity.

Analysis of microsatellite data (STRUCTURE) identified three clusters in the African lion: 1) West/Central Africa, 2) East Africa, and 3) Southern Africa, in addition to a cluster comprising the Asiatic subspecies. Although the high level of fixation of alleles in the Asiatic lion is likely to contribute to the identification of this population as a distinct cluster, genetic structure is found within the African subspecies. This supports the genetically distinct position of lions from West/Central Africa reported previously and found again here based on mtDNA data [2,3,9]. In addition, STRUCTURE also indicates divergence within the East and Southern African lions. The observed split between East and Southern Africa, as was previously shown by Bruche *et al*. (2012), remained after inclusion of a population from Zambia which is geographically intermediate between Tanzania and RSA [12]. Bruche *et al*. (2012) concluded that the Ethiopia1 population forms a unique clade, as it showed to be distinct from India, East Africa and Southern Africa [12]. In this study, we describe that Ethiopia1 shows strong admixture with West/Central Africa based on microsatellite data, which is further confirmed by the mitochondrial haplotype. This leads to the conclusion that these individuals do not form a unique group, but are instead assigned for a substantial part to a cluster that was not represented in the work by Bruche *et al*. (2012) [12]. Although the origin of the Ethiopia1 founder lions is disputed, it is claimed that they originate from the south-western part of Ethiopia [46] west of the Rift Valley, which has previously been suggested as a barrier for lion dispersal [4,5,9,47,48]. The other captive Ethiopian population, Ethiopia2, contains a haplotype that clusters within the East Africa group. Assessment of the microsatellite data showed that Ethiopia2 individuals indeed contained a stronger signal from East Africa, compared to Ethiopia1. The observed admixture in both captive Ethiopian lion populations may be explained by the geographical location of Ethiopia, however, human-mediated translocations are not uncommon in zoo settings and may have contributed to the observed pattern. In Zambia, a substructure in the population is induced due to the two detected types of admixture: the Southern Africa cluster is admixed either with the West/Central Africa cluster, or with the East Africa cluster. These findings are parsimonious with the geographic isolation representative of Zambia’s Luangwa Valley which is an offshoot of the Rift Valley System. The absence of a mitochondrial haplotype from outside the East Africa cluster in the Zambian individuals indicates that the pattern of admixture is likely due to male-mediated gene flow.

The mtDNA haplotype network shows the same four groups as identified in the STRUCTURE analysis: 1) West/Central Africa, 2) East Africa, 3) Southern Africa and 4) India. The deepest split in the haplotype network distinguishes a North group consisting of the West/Central African lion together with the Asiatic subspecies, and a South group consisting of lions from East and Southern Africa. Within a single country, only one or two closely related haplotypes are found, with two exceptions where more divergent haplotypes are present: 1) Ethiopia, which could be explained by the geographic location of the country as previously noted, and 2) RSA2, likely due to past translocations to and amongst small reserves in RSA [49]. The neighbour-joining tree, based on microsatellite data also shows a distinction between lions from West/Central Africa, and populations from East and Southern Africa. The basal position of the Indian and Kenyan lions probably results from the lower genetic diversity in these populations, as is indicated by the relatively high number of monomorphic loci. Elongation of branch length resulting from a population size reduction has been previously described, especially for D_A_ as a measure of genetic distance [50]. Despite of this, D_A_ is commonly accepted as the most suitable measure for inferring phylogenetic relationships [51,52] and, therefore, has been used in our analyses. STRUCTURE and PCA plots show that all populations from Namibia and RSA are assigned to Southern Africa, with a more central position for the admixed Zambia population, while East African haplotypes are found in RSA. The same discrepancy was previously described by Antunes *et al*. (2008) and attributed to sex-biased gene flow. To further assess congruence between mtDNA and autosomal markers, a Mantel test was performed based on corrected Nei’s genetic distances for both datasets (S8 Table). This illustrates a significant relationship (999 permutations, P<0.01) between both measures, which increases further after the exclusion of India. Strongest congruence in AMOVA results between the autosomal and mtDNA data are found when using the groups identified by STRUCTURE, indicating a robust phylogenetic pattern that is reflected by both genetic markers.

The four lineages we describe based on autosomal and mtDNA data are further corroborated by the distinction of four groups based on morphological data [7]. Up to eight “subspecies” have been used by some sources [53], with the Barbary lion (*P*. *l*. *leo*) very likely to be extinct and the Cape lion (*P*. *l*. *melanochaita*) a possible con(sub)specific with *P*. *l*. *krugeri* [10]. Of the remaining six subspecies, Hemmer (1974) suggests to not include *P*.*l*.*bleyenberghi* (South West Africa) and *P*.*l*.*azandica* (North East DRC) as fully differentiated lineages. The remaining four subspecies, *P*. *l*. *persica* and *P*. *l*. *senegalensis* in the northern part of the range, and *P*. *l*. *nubica* and *P*. *l*. *krugeri* in the southern part of the range correspond to India, West/Central Africa, East Africa, and Southern Africa respectively, and reflect the deepest split in the haplotype network. Although sample size was limited, the close genetic relationship of West/Central African lions to the Asiatic subspecies, was later reconfirmed by craniometric data [8].

IBD explains the genetic distances on a continental scale and on a regional scale. The strong slope of the trend line in IBD analysis for West/Central Africa, compared to Southern and East Africa, is suggestive of near complete isolation between populations in the West/Central region. This is also supported by the high among-groups variance in the AMOVA. Based on the genetic distances (pairwise F_ST_ and Nei’s genetic distance), we conclude that all sampled populations are significantly differentiated from each other.

It was hypothesized that lion populations in West Africa and parts of Central Africa were especially vulnerable to declining levels of genetic diversity since fragmentation of the habitat is particularly severe in this region. However, we did not find significant heterozygotic deficiencies, reduced number of alleles or fixed loci in any of the six sampled populations in this region. The significantly negative F_IS_ values (excess of heterozygotes) may be explained by the mating system as was also shown for prides in Selous GR [31], however we acknowledge the possible effect of a small sample size in our study. The unexpectedly high levels of genetic diversity could further be explained by the fact that the range contraction and the decline of lion numbers is too recent to show clear signs of genetic erosion. However, because genetic diversity is rapidly lost in small populations as a result of genetic drift and inbreeding, keeping the population at a genetically healthy level may require urgent management decisions to safeguard against these effects. Monitoring of an intensively managed lion population showed that drift and inbreeding were noticeable within five years after reintroduction of eleven founders from four genetic lineages [54]. The strongly significant heterozygote deficiency observed in the Zambia lion population is likely to be the result of substructure in the population (Wahlund effect), which was consistent with the results from the STRUCTURE analysis. The significantly positive F_IS_ value found in RSA1 is congruent with previous findings [4] and a high F_IS_ value in the Ethiopia1 lions can be explained by the breeding history of the population, which was founded by five males and two females in 1948 [12]. In addition, both RSA1 and Ethiopia1 were indicated by Bottleneck to have gone through recent population reductions. Similarly, Cameroon1 and Kenya appear to have experienced bottlenecks, which is consistent with observations obtained from monitoring studies [1,55], although we cannot completely rule out the effect of low sample sizes. Since the excess of heterozygotes as a result of a bottleneck is transient, the Bottleneck approach only detects recent reductions in population size, which explains why historically documented bottlenecks i.e., Tanzania2 and India, were not detected. Our study is the first to confirm that autosomal markers support the distinct genetic position of West/Central African lions within the African subspecies. The phylogenetic split between West/Central Africa and East/Southern Africa found in other species is reiterated in lions. Based on results derived from mtDNA data and from autosomal microsatellites, we recommend recognition and consideration of these four groups for management decisions: 1) West/Central Africa, 2) East Africa, 3) Southern Africa and 4) India. In consideration of genetic distinctions coupled with anthropogenic factors that are accelerating decline of wildlife in West and Central Africa, this region is of particular and urgent conservation importance. By showing a congruent phylogeographic pattern in both mtDNA and autosomal markers, our data illustrate which populations belong to the same evolutionary lineage and may contribute importantly to conservation decisions e.g., identifying suitable candidates for translocations or population augmentation. We support a revision of the taxonomic nomenclature as has been proposed by Barnett *et al*. (2014), following the deepest ancestral split found in the haplotype network, recognizing a North group and a South group. Primarily, as mtDNA, autosomal markers and morphological data show a congruent pattern, we believe that it is enough to support a taxonomic split within the African subspecies of the lion.

## Supporting Information

S1 File Details on sample storage, DNA extraction, PCR, fragment analysis and sequencing. (DOCX)

S2 File Microsatellite allele length for 20 loci in 16 lion populations. (TXT)

S3 File Details on DNA extraction, PCR, fragment analysis for Dataset 3. (DOCX)

S4 File Mitochondrial haplotype sequences for 16 lion populations. (FAS)

S1 Table Overview of microsatellite loci used in the different lion populations. (XLSX)

S2 Table Primers used for amplification of microsatellites and mtDNA. (XLSX)

S3 Table Identified haplotypes and accompanying accession numbers from Genbank. (XLSX)

S4 Table Results of an AMOVA for a microsatellite dataset of 12 lion populations and a mtDNA dataset of 16 lion populations. (XLSX)

S5 Table Results of the Mantel tests indicating IBD effects in lion populations on a continental and regional scale. (XLSX)

S6 Table Pairwise F_ST_ (below diagonal) and Nei's genetic distances (above diagonal) based on 20 microsatellite loci from 14 lion populations. (XLSX)

S7 Table Genetic variation in microsatellite loci and mtDNA among 16 lion populations. (XLSX)

S8 Table Nei's (corrected) genetic distances for microsatellite (below diagonal) and mtDNA data (above diagonal) of 14 lion populations and results of Mantel tests including all populations and excluding India. (XLSX)
