# Correction: Autosomal and mtDNA Markers Affirm the Distinctiveness of Lions in West and Central Africa

**DOI:** 10.1371/journal.pone.0149059

**Published:** 2016-03-03

**Authors:** Laura D. Bertola, Laura Tensen, Pim van Hooft, Paula A. White, Carlos A. Driscoll, Philipp Henschel, Anthony Caragiulo, Isabela Dias-Freedman, Etotépé A. Sogbohossou, Pricelia N. Tumenta, Tuqa H. Jirmo, Geert R. de Snoo, Hans H. de Iongh, Klaas Vrieling

In Table 1, the information in the “Source msat data” column is incorrect for the “Zambia” row. Please see the corrected Table 1 here.

**Table 1:** Overview of lion populations included in this study.

Set	Population	Area	Geographic Region	PopSize	N msat	N mtDNA	Source msat data
	**Benin**	Pendjari NP	West Africa	100	5	5	this dataset
	**Cameroon1**	Waza NP	Central Africa	20	9	9	this dataset
	**Cameroon2**	Bénoué Ecosystem	Central Africa	200	3	3	this dataset
	**Chad**	Zakouma NP	Central Africa	140	4	4	this dataset
	**DRC**	Garamba NP	Central Africa	175	7	6	this dataset
	**Ethiopia2**	Yemen Zoo	East Africa	(captive)	4	4	this dataset
**1**	**Kenya**	Amboseli NP	East Africa	60	7	7	this dataset
	**Tanzania1**	Serengeti NP	East Africa	3465	10	3	Driscoll et al., 2002
	**Tanzania2**	Ngorongoro CA	East Africa	53	10	1	Driscoll et al., 2002
	**Zambia**	Luangwa Valley	Southern Africa	750	9	9	this dataset
	**Namibia**	Etosha NP	Southern Africa	455	10	2	Driscoll et al., 2002
	**RSA1**	Kalahari-Gemsbok NP	Southern Africa	350	10	2	Driscoll et al., 2002
	**RSA2**	Kruger NP	Southern Africa	1684	10	10*	Driscoll et al., 2002
	**India**	Gir forest NP	India	411	10	6	Driscoll et al., 2002
**2**	**Ethiopia1**	Addis Ababa Zoo	East Africa	(captive)	15	5	Bruche et al., 2012
**3**	**Senegal**	Niokolo Koba NP	West Africa	15	7	7	Panthera/AMNH

PopSize: population size according to the most recent estimate in Riggio et al. (2012) for the African populations, except for Zambia: Paula White (personal communication); estimate for the Indian population from [56]

N msat: number of sampled individuals for microsatellite analysis

N mtDNA: number of sampled individuals for mtDNA analysis.

* mtDNA and microsatellite data are not from the same samples.
