# Supplementary material for: Autosomal and mtDNA Markers Affirm the Distinctiveness of Lions in West and Central Africa
Source: PLoS One. 2015 Oct 14;10(10):e0137975. doi: 10.1371/journal.pone.0137975 (PMC4605676; doi:10.1371/journal.pone.0137975)
Supplement: S1 File — (DOCX) [file pone.0137975.s001.docx]

**Supplemental Information 1: Details on sample storage, DNA extraction, PCR, fragment analysis and sequencing.**

**Permits**

No samples were collected specifically for this study, and all included samples had been collected during previous fieldwork and stored in biobanks. More detailed information about permits and issuing authorities for each included population is given below:

Benin: Direction Generale des Forets et Ressources Naturelles - DGFRN (National Directorate for Forests and Natural Resources), local park authorities Pendjari Biosphere Reserve: samples were collected during previous fitting of GPS collars on lions (publication in prep. by Sogbohossou et al.), no individuals were handled for this study.

Cameroon: Ministry of Environment and Forestry, local park authorities Waza National Park and Bénoué Ecosystem: samples were collected during previous fitting of GPS collars on lions (publication by Tumenta et al., 2009), no individuals were handled for this study.

Chad: Ministry of Environment, Water, & Fisheries, local park authorities Zakouma National Park: samples were collected during previous studies, no individuals were handled for this study.

DRC: Institut Congolais pour la Conservation de la Nature - African Parks Network, Garamba National Park management: samples were collected during previous fitting of GPS collars on lions, no individuals were handled for this study.

Zambia: Zambia Wildlife Authority (ZAWA): samples were collected during previous studies (publication by Dubach et al. (2013)), no individuals were handled for this study.

RSA: South African National Parks (SANParks): samples were collected during previous studies, no individuals were handled for this study.

Kenya: Kenya Wildlife Service (KWS): samples were collected during previous fitting of GPS collars on lions (publication in prep. by Jirmo et al.), no individuals were handled for this study.

Ethiopia (captive), Tanzania, Namibia, RSA: samples were obtained from the IZW Leibniz Institute for Zoo and Wildlife Research (Germany). Samples were collected during previous studies (publications by Driscoll et al. (2002) and Bruche et al. (2012)), no individuals were handled for this study.

India: samples were obtained from National Cancer Institute (NCI) (U.S.A.): samples were collected during previous studies (publication by Driscoll et al., 2002), no individuals were handled for this study.

**Sample storage and DNA extraction**

Blood and tissue samples were preserved dried (Zambia) or in buffer (0.15 M NaCl, 0.05 M Tris-HCl, 0.001 M EDTA, pH = 7.5) and stored at -20°C. DNA was extracted using the DNeasy Blood & Tissue kit (Qiagen) following the manufacturer’s protocol.

**Microsatellite analysis**

Twenty microsatellite loci, originally developed for domestic cat (Menotti-Raymond *et al.* 1999), were selected from a set that had previously been used in lion (Driscoll *et al.* 2002) (see Supplemental Table 2). A selection was made based on high variability, high amplification success and no apparent presence of null alleles. To enable resizing to already published datasets from Driscoll *et al.* (2002) and Bruche *et al.* (2012), four samples of these studies (Tanzania10 and RSA10; Ethiopia12 and Ethiopia13) were included for all 20 microsatellites. During PCR the products were fluorescently labelled (HEX, TAMRA and FAM) by adding M13 tails to the 5’end (Schuelke 2000). PCR reactions contained 0.75 mM MgCl_2_, 0.4 mg/ml bovine serum albumin, 10x PCR buffer, 200 μM dNTPs, 0.1 U/μl Taq polymerase, 0.4 μM of both amplification primers and the M13 fluorescently labeled primer, and 1 μl of DNA template in a total volume of 15 μl. The PCR reaction was performed using an initial denaturation step of 94°C for 4min, followed by 40 cycles of 94°C for 20s, 51°C for 1 min, 72°C for 1 min and a final extension step of 72°C for 10 min. PCR products with different labels and non-overlapping fragment sizes (min. 20 bp difference between longest and shortest allele documented) were pooled and run on a MegaBACE sequencer (GE Health Care, Eindhoven, The Netherlands) or ABI3730XL (Macrogen Inc., Amsterdam, The Netherlands) with ET-ROX400 or ROX400 as an internal standard. To enable comparisons between runs and machines, we included a minimum of two known samples for every locus in each run. Allele lengths were scored using MegaBace Fragment Profiler version 1.2 (Amersham Biosciences, 2003) or Peak Scanner Software v1.0 (Life Technologies). Samples with a weak or distorted signal were re-amplified and were included in a subsequent run.

Microsatellite data were checked for potential null alleles and allelic dropout using Microchecker (Van Oosterhout *et al.* 2004). The data were tested for linkage disequilibrium using the Fisher’s exact test in GENEPOP 4.2.1 (Raymond & Rousset 1995), applying 10000 dememorisations, 100 batches and 5000 iterations per batch as Markov chain parameters.

**mtDNA analysis**

PCRs were performed with three primer pairs (Supplemental Table 2), designed with the web-based software Primer3v 0.4.0 (Rozen & Skaletsky 2000). PCR reactions contained 1 mM MgCl_2_, 0.4 mg/ml bovine serum albumin, 10x PCR buffer, 200 μM dNTPs, 0.1 U/μl Taq polymerase, 0.4 μM of both amplification primers and 1 μl of DNA template in a total volume of 20 μl. The PCR program consisted of an initial denaturation step of 94°C for 4min, followed by 40 cycles of 94°C for 20s, annealing temperature ranging from 51°C to 54°C depending on the primer set, for 1 min, 72°C for 1 min and a final extension step of 72°C for 10 min. Sequencing was performed by Macrogen Inc., Amsterdam, The Netherlands.

**Quality control**

Out of a total of 2188 data points (Dataset 1: 104 samples * 20 loci + 4 samples (Chad) * 15 loci + 4 samples (Ethiopia2)* 12 loci), Dataset 1 included a total of 28 missing genotypes (1.28%). None of the individuals had missing values at more than two loci. Indications for stuttering errors or null-alleles, as is suggested by the general excess of homozygotes in Microchecker, were identified in three populations for one locus (FCA178 in Ethiopia1 and Namibia; FCA211 in RSA1) and in the Zambia population for six loci (FCA026, FCA057, FCA094, FCA208, FCA211 and FCA224). However, in the case of the Zambia population this is likely to be the result of genetic structure within the population (see results STRUCTURE analysis). Since none of the loci were consistently positive for more than two populations, we included all loci in downstream analyses. There was no indication of allelic dropout. Pairwise comparison of loci in each population identified significant linkage (P<0.05) in 37 cases in a total of 2850 comparisons (1.30%). No loci were consistently in linkage disequilibrium across populations and a pairwise comparison between loci on the entire dataset did not reveal significant linkage.

**References**

Driscoll CA, Menotti-Raymond M, Nelson G, Goldstein D, O’Brien SJ (2002) Genomic microsatellites as evolutionary chronometers: a test in wild cats. *Genome research*, **12**, 414-423.

Menotti-Raymond M, David V, Lyons LA *et al.* (1999) A genetic linkage map of microsatellites in the domestic cat (Felis catus). *Genomics*, **57**, 9-23.

Van Oosterhout C, Hutchinson WF, Wills DPM, Shipley P (2004) Micro-Checker: Software for Identifying and Correcting Genotyping Errors in Microsatellite Data. *Molecular Ecology Notes*, **4**, 535-538.

Raymond M, Rousset F (1995) GENEPOP (version 1.2): Population genetics software for exact tests and ecumenicism. *Journal of Heredity*, 248-249.

Rozen S, Skaletsky HJ (2000) Primer3 on the WWW for general users and for biologist programmers. In: *Bioinformatics Methods and Protocols: Methods in Molecular Biology* (eds S. K, S. M), pp. 365-386. Humana Press, Totowa, NY.

Schuelke M (2000) An economic method for the fluorescent labeling of PCR fragments. *Nature Biotechnology*, **18**, 233-234.
