# Supplementary material for: Autosomal and mtDNA Markers Affirm the Distinctiveness of Lions in West and Central Africa
Source: PLoS One. 2015 Oct 14;10(10):e0137975. doi: 10.1371/journal.pone.0137975 (PMC4605676; doi:10.1371/journal.pone.0137975)
Supplement: S3 File — (DOCX) [file pone.0137975.s003.docx]

**Supplemental Information 3: Details on DNA extraction, PCR, fragment analysis for Dataset 3.**

**Permits**

Senegal: No specific permits were required for the collection and export of samples from Senegal. The samples were collected as part of a lion survey carried out jointly with the Senegalese national park authority (Direction des Parcs Nationaux; DPN). DPN waived all permit requirements, to facilitate the timely assessment of lion population status in the park, including of its genetic make-up. Samples were collected non-invasively (scat), no individuals were handled for this study.

**DNA extraction & species identification**

Genomic DNA was extracted from scat samples using the QIAmp DNA Stool Mini Kit (QIAGEN, Valencia, California, USA) following the manufacturer’s protocol for isolation from stool for human DNA analysis with some modifications to improve DNA quality and quantity. All instruments were cleaned with DNAaway™ (Molecular BioProducts, San Diego, California, USA) and placed in an ultraviolet cross-linker prior to use. Additionally, all extractions were performed on a dedicated lab bench and in a pre-PCR laboratory to avoid contamination. Two microcentrifuge tubes were prepared with approximately 180 – 200 mg per sample in each using shavings from the outer layer of the scat sample. Samples were incubated overnight (approximately 18 h) at 56 °C on a rotator (approximately 22 RPM) with 1.5 mL of ASL buffer. All of the supernatant for each unique sample was combined into two tubes, with one tube containing 1.5 mL of supernatant and the other tube having all remaining supernatant up to 1.5 mL. A proportionate amount of InhibitEx tablet was added to the second tube of supernatant if the volume was less than 1.5 mL. Cold ethanol was used and AE elution buffer was heated to 70°C prior to addition to the spin-column membrane. Elution of DNA from the spin-column membrane was carried out in three stages, with the addition of 60 μL of AE buffer each time and a 40 minute and two 15 minute incubations at room temperature prior to centrifugation, for a total elution volume of 180 μL.

All scat samples were screened for species identification using species-specific primers amplifying regions of four mitochondrial genes as described in Caragiulo *et al.* (2013) (Table 1). DNA amplifications from all extractions were carried out in 25 μL reaction volumes containing 22.3 μL of ultrapure water, 0.7 μL of forward primer (10 μM), 0.7 μL of reverse primer (10 μM), 0.3 μL bovine serum albumin (BSA), one illustra™ puReTaq Ready-To-Go PCR Bead (GE Healthcare, Piscataway, New Jersey, USA), and 1.0 μL of template DNA.

All samples were visualized on a 2.0% agarose gel in TBE buffer and PCR products were purified using the Agencourt AMPure XP PCR purification protocol (Beckman Coulter, Indianapolis, Indiana, USA). Purified PCR products were then sequenced using the Big Dyeterminator protocol (Applied Biosystems, Carlsbad, California, USA). Sequencing reactions were carried out in 8.0 μL reaction volumes containing 4.75 μL ultrapure water, 0.75 μL extension buffer, 0.5 μL Big Dye, 1.0 μL primer (1.6 μM), and 1.0 μL of purified PCR product. The sequencing reaction was carried out separately for the forward and reverse primers. The thermocycler profile for all sequencing reactions followed that of Platt *et al.* (2007). Sequencing amplifications were purified using the Agencourt CleanSEQ Dye Terminator Removal protocol (Beckman Coulter, Indianapolis, Indiana, USA) and sequenced in an ABI 3730xl DNA Analyzer (Applied Biosystems, Carlsbad, California, USA). Sequences were manually edited using Sequencher (version 5.0, Gene Codes Corporation, Ann Arbor, Michigan, USA) and Geneious (Drummond *et al.*, 2012), and compared to both an in-house database of carnivore mtDNA sequences and the NCBI nucleotide BLAST database to confirm species identification. All samples identified as lion were used in further analyses.

The 12 microsatellite loci used in this study (FCA032, FCA075, FCA096, FCA100,FCA124, FCA126, FCA132, FCA208, FCA212, FCA225, FCA229, FCA275) were adapted from a genetic map of the domestic cat (Menotti-Raymond *et al.*, 1999) and optimized for lions. PCR reactions were carried out in 20.0 μL multiplex reactions containing 5 μL of extracted DNA, 0.20 – 1.60 μL of each forward and reverse 10 μM primer,10.0 μL Qiagen Mutliplex PCR Master Mix, 2.0 μL Q-solution, and the remaining volume was RNAse-Free water (Qiagen, Valencia, California, USA). Primers were grouped into five multiplex reactions based upon fluorescent tag and amplicon size (Table 2). Thermocycling conditions were the same for each multiplex, except for the touchdown and annealing temperature, and were as follows: 95 °C for 15 minutes, 13 cycles of 94 °C for 30 seconds, touchdown annealing temperature for 1.5 minutes, and 72 °C for 1 minute, followed by 32 cycles of 94 °C for 30 seconds, annealing temperature for 1.5 minutes, and 72 °C for 1 minute, followed by 30 minutes at the annealing temperature (Table 2). Samples were prepared for analysis by mixing 1 μL of PCR product with 9 μL of an 8.82 μL : 0.18 μL mixture of Hi-Di formamide: GeneScan 500 LIZ size standard (Applied Biosystems, Carlsbad, California, USA). Samples were heat-shocked for 3 minutes at 95 °C and genotypes were analyzed using an ABI 3730xl DNA analyzer (Applied Biosystems, Carlsbad, California, USA). Genotypes were scored with GeneMapper v. 4.0 software (Applied Biosystems, Carlsbad, California, USA) and individually verified by visual inspection.

All microsatellite amplifications were performed at least four times using the multi-tube approach (Taberlet *et al.*, 1996) to identify possible allelic dropout. Allelic dropout and PCR success was quantified per locus using GIMLET (Valière, 2002). Consensus genotypes were defined for each sample by comparing results from both a consensus genotype inference method using GIMLET (Valière, 2002) and manual inspection. All samples that did not produce reliable consensus genotypes for at least 6 loci were excluded from further analyses.

**References**

1. Benson, D.A., Karsch-Mizrachi, I., Lipman, D.J., Ostell, J. & Wheeler, D.L. 2005 GenBank. *Nucleic Acids Research*, **33**, D34-D38.
2. Caragiulo, A., Dias-Freedman, I., Clark, J.A., Rabinowitz, S. & Amato, G. 2013 Mitochondrial DNA sequence variation and phylogeography of Neotropic pumas (Puma concolor). *Mitochondrial DNA*, **0**, 1-9.
3. Chaves P.B., Graeff V.G., Lion M.B., Oliveira L.R., Eizirik E. 2012 DNA barcoding meets molecular scatology: Short mtDNA sequences for standardized species assignment of carnivore noninvasive samples. *Molecular Ecology Resources*, **12**, 18–35.
4. Drummond, A., Ashton, B., Buxton, S., Cheung, M., Cooper, A., Heled, J., Kearse, M., Moir, R., Stones-Havas, S., Sturrock, S., Thierer, T. & Wilson, A. 2012 Geneious v5.6.
5. Farrell, L., Roman, J. & Sunquist, M.E. 2000 Dietary separation of sympatric carnivores identiﬁed by molecular analysis of scats. *Molecular Ecology*, **9**, 1583-1590.
6. Kitano T., Umetsu K., Tian W., Osawa M. 2007 Two universal primer sets for species identification among vertebrates. *International Journal of Legal Medicine*, **121**, 423–7.
7. Kocher, T.D., Thomas W.K., Meyer A. et al. 1989 Dynamics of mitochondrial DNA evolution in animals: amplification and sequencing with conserved primers. *Proceedings of the National Academy of Sciences USA*, **86**, 6196-6200.
8. Menotti-Raymond, M., David, V., Lyons, L., Schäffer, A., Tomlin, J., Hutton, M. & O'Brien, S. 1999 A genetic linkage map of microsatellites in the domestic cat (*Felis catus*). *Genomics*, **57**, 9-23.
9. Paxinos E., McIntosh C., Ralls K. & Fleischer R. 1997 A noninvasive method for distinguishing among canid species: amplification and enzyme restriction of DNA from dung. *Molecular Ecology*, **6**, 483-486.
10. Pomilla C., Harmsen B.J., Foster R.J., Devlin A., Bartholomew C., Vynne C., Carillo-Percastegui S., et al. 2009 Large and fine scale genetic characterization of jaguars (Panthera onca) throughout their range. Oral Presentation at the 23rd International Congress for Conservation Biology, July, Beijing, China.
11. Taberlet, P., Griffin, S., Goossens, B., Questiau, S., Manceau, V., Escaravage, N., Waits, L.P. & Bouvet, J. 1996 Reliable genotyping of samples with very low DNA quantities using PCR. *Nucleic Acids Research*, **24**, 3189-3194.
12. Valière, N. 2002 GIMLET: a computer program for analysing genetic individual identification data. *Molecular Ecology Notes*, **2**, 377-379.
13. Wei, K., Zhang, Z., Zhang, W., Xu, X., Liang, X., He, G., Shen, F., Zhang, L., Hou, R. & Yue, B. 2008 PCR-CTPP: a rapid and reliable genotyping technique based on ZFX/ZFY alleles for sex identification of tiger (*Panthera tigris*) and four other endangered felids. *Conservation Genetics*, **9**, 225-228.

**Table 1. Four mitochondrial gene regions spanning 1,140 bp were amplified using the primer sets described below. The Carnivorous primers amplify a region nested within the region amplified by the Canideos primers.**

| **Type** | **Location** | **Primername** | **Annealing Temp (˚C)** | **Origin** |
| --- | --- | --- | --- | --- |
| mtDNA | Cytochrome b | Carnivorous F: H15149 | 50 | Kocher et al. 1989 |
|  |  | Carnivorous R: Farrel-R |  | Farrell et al. 2000 |
|  |  | Canideous F: H15149 | 52 | Kocher et al. 1989 |
|  |  | Canideos R: Canid-L1 |  | Paxinos et al. 1997 |
|  | 12S rRNA | L1085 | 57 | Kitano et al. 2007 |
|  |  | H1259 |  | Kitano et al. 2007 |
|  | 16S rRNA | 16Scp-F | 52.5-51.5 (touchdown cycle) | Pomilla et al. 2009 |
|  |  | 16Scp-R |  | Pomilla et al. 2009 |
|  |  | L2513 | 57 | Kitano et al. 2007 |
|  |  | H2714 |  | Kitano et al. 2007 |
|  | ATPase-6 | ATP6-DF3 | 50 | Chaves et al. 2012 |
|  |  | ATP6-DR2 |  | Chaves et al. 2012 |

**Table 2. FCA primers were grouped into five multiplexes and thermocycling conditions were optimized for each multiplex.**

| **Group** | **Included loci** | **Touchdown Cycle Annealing Temperature (˚C)** | **Annealing Temp (˚C)** |
| --- | --- | --- | --- |
| **Multiplex 1** | FCA032 | 60.4 - 0.3 | 58 |
|  | FCA100 |  |  |
|  | FCA124 |  |  |
| **Multiplex 2** | FCA126 | 62.4 - 0.3 | 60 |
|  | FCA212 |  |  |
|  | FCA229 |  |  |
| **Multiplex3** | FCA096 | 59.4 - 0.3 | 57 |
|  | FCA132 |  |  |
|  | FCA275 |  |  |
| **Multiplex 4** | FCa075 | 59.4 - 0.3 | 57 |
|  | FCA208 |  |  |
| **Multiplex 5** | FCA225 | 57.4 - 0.3 | 55 |
